# Identification and Characterization of Wheat Germplasm for Salt Tolerance

**DOI:** 10.3390/plants10020268

**Published:** 2021-01-30

**Authors:** Xiaoyan Quan, Xiaoli Liang, Hongmei Li, Chunjuan Xie, Wenxing He, Yuxiang Qin

**Affiliations:** School of Biological Science and Technology, University of Jinan, Jinan 250022, China; bio_quanxy@ujn.edu.cn (X.Q.); 13205410860@163.com (X.L.); chm_lihm@ujn.edu.cn (H.L.); 19861428869@163.com (C.X.)

**Keywords:** wheat, salinity, salt tolerance, osmotic substance, antioxidative stress

## Abstract

Salinity is one of the limiting factors of wheat production worldwide. A total of 334 internationally derived wheat genotypes were employed to identify new germplasm resources for salt tolerance breeding. Salt stress caused 39, 49, 58, 55, 21 and 39% reductions in shoot dry weight (SDW), root dry weight (RDW), shoot fresh weight (SFW), root fresh weight (RFW), shoot height (SH) and root length (RL) of wheat, respectively, compared with the control condition at the seedling stage. The wheat genotypes showed a wide genetic and tissue diversity for the determined characteristics in response to salt stress. Finally, 12 wheat genotypes were identified as salt-tolerant through a combination of one-factor (more emphasis on the biomass yield) and multifactor analysis. In general, greater accumulation of osmotic substances, efficient use of soluble sugars, lower Na^+^/K^+^ and a higher-efficiency antioxidative system contribute to better growth in the tolerant genotypes under salt stress. In other words, the tolerant genotypes are capable of maintaining stable osmotic potential and ion and redox homeostasis and providing more energy and materials for root growth. The identified genotypes with higher salt tolerance could be useful for developing new salt-tolerant wheat cultivars as well as in further studies to underline the genetic mechanisms of salt tolerance in wheat.

## 1. Introduction

As one of the most significant abiotic stresses influencing grain yield, salinity has become a great threat to agricultural sustainability, affecting more than 800 million hectares of land around the world [1,2,3]. Development of salt-tolerant cultivars is one of the most effective ways to deal with this issue [4]. The prerequisite is to identify genotypes with wide adaptation to salinity. Nevertheless, few identified wheat genotypes with salt tolerance have been widely used in breeding for salt-tolerant cultivars so far [4,5]. Therefore, new germplasms with salt tolerance need to be identified to broaden the gene base and to provide elite resources with widely adapted genetic backgrounds.

Salt stress damages plants through osmotic stress, ionic toxicity and oxidative stress in cells [6,7,8]. Consequently, plants have developed various mechanisms for salinity stress adaptation or tolerance. Plants enhance their tolerance to osmotic stress by maintaining cell turgor through the accumulation of soluble osmotic adjustment substances such as proline and soluble sugars [9,10,11]. Meanwhile, tissue tolerance to ionic toxicity can be improved by regulating Na^+^/K^+^ homeostasis and Na^+^ compartmentalization and exclusion [12,13,14]. In addition, antioxidant protection systems may be stimulated to scavenge reactive oxygen species (ROS), resulting in the enhancement of tolerance to oxidative stress [15,16]. Malondialdehyde (MDA) is well known to be a product of membrane lipid peroxidation caused by the increase of ROS under salt stress, and it is considered an indicator of cell membrane damage [10]. Thus, the content of proline, soluble sugars and MDA, the Na^+^/K^+^ value and antioxidant enzyme activities have been used as physiological indicators for stress tolerance evaluation in many studies [10,17,18].

Screening wheat genotypes with salt tolerance has been inhibited by the lack of effective evaluation methods [2,4,19]. It is quite imperative to determine growth or physiological parameters as the tolerant index for the evaluation of abiotic stress tolerance [4,20,21]. The most valuable agronomic traits might serve as good indicators to distinguish among genotypes under salt stress. Biomass yield is considered a useful indicator because it permits the direct estimation of economic return under salt stress [22]. The salt tolerance status of plants has been evaluated by the ratio of biomass in saline and control conditions, and there has been some success [23]. However, additional valuable agronomic traits should be taken into consideration, as salt tolerance is a complex biological trait governed by several physiological and genetic factors [10,20]. In addition, plant salt tolerance is also growth stage-specific, and the salt sensitivity of physiological traits varies at different growth stages [24]. This makes the selection of salt-tolerant indicators very difficult and leads to low selection efficiency [4,24]. To date, numerous physiological traits have been evaluated as suitable for screening tolerant genotypes [10,20]. Despite the low selection efficiency using overall agronomic parameters, several studies have succeeded in assessing salt tolerance through multivariate analysis in wheat [4,25,26]. Nevertheless, the methods for ranking genotypes still need to be improved when multiple salinity-sensitive agronomic characteristics are used as indicators simultaneously. Agronomic characteristics are always primary targets in plant breeding, and yield is of the most concern in agricultural production. Taking all the foregoing information into account, we believe that a combination of biomass ranking and integrated scoring of multiple agronomic traits will contribute to the effective evaluation of salt-tolerant genotypes.

As one of most important crops worldwide, wheat (*Triticum aestivum* L.) suffers significant yield reduction due to soil salinity. A better understanding of the mechanisms underlying salt tolerance would be of significance for the breeding of salt-tolerant cultivars. The present investigation was undertaken to characterize salt tolerance in 334 wheat genotypes from around the world at the seedling stage, aiming to identify salt-tolerant genotypes through a combination of one-factor and multifactor analysis. The effects of salt stress on some key physiological traits, including leaf chlorophyll content, cell membrane stability, osmotic adjustment substances and antioxidant enzyme activities, were evaluated in seven genotypes with contrasting tolerance (four highly tolerant, one moderately tolerant and two sensitive). The identified genotypes may be excellent material candidates for cultivation and for a better understanding of the mechanisms of salt tolerance in wheat.

## 2. Results

### 2.1. Genotypic Differences in Response to Salt Stress

In the screening experiment, wheat plant growth was markedly inhibited by salt stress for all accessions. On average, shoot dry weight (SDW), root dry weight (RDW), shoot fresh weight (SFW), root fresh weight (RFW), shoot height (SH) and root length (RL) of wheat subjected to salt stress were reduced by 39, 49, 58, 55, 21 and 39%, respectively, compared with the control condition (Table 1 and Table S1). Additionally, 334 wheat accessions showed a wide range of variation in SFW, RFW, SDW, RDW, SH, RL and their ratios under salt stress relative to those of the control (designated as RSFW, RRFW, RSDW, RRDW, RSH and RRL, respectively) (Table 1). Among the wheat genotypes, the highest coefficient variation (CV) was for RRFW, and the lowest was for RSH (Table 1).

Among all the accessions, SFW ranged from 660 to 2070 mg and from 232 to 810 mg under the control condition and salt stress, respectively, while RFW ranged from 502 to 1759 mg under the control condition and from 131 to 825 mg under salt stress (Table 1). The salt/control value ranged from 0.22 (Yangmai15) to 0.78 (Xinong291) for SFW and from 0.14 (HK1/6/NVSR3/5/BEZ/TVR/5/CFN/BEZ//SU92/CI13645/3NAI60) to 1.05 (Xinong291) for RFW, respectively (Table 1).

For dry weight, SDW ranged from 84 to 214 mg under the control condition and from 43 to 130 mg under salt stress, while RDW ranged from 33 to 105 mg and from 9 to 51 mg under the control condition and salt stress, respectively (Table 1). The salt/control value ranged from 0.37 (Chuanmai107) to 0.94 (Taishan24) for SDW and from 0.17 (Chuanmai107) to 1.00 (Xinong291) for RDW, respectively (Table 1). In generally, the variation trend of the dry weights of shoots and roots was basically consistent with that of fresh weight values.

For other growth parameters, SH ranged from 17 to 27 cm under the control condition and from 12 to 23 cm under salt stress, while RL was from 24 to 49 cm and from 8 to 30 cm under the control condition and salt stress, respectively (Table 1). The salt/control value ranged from 0.62 (Libero) to 1.03 (Xinong291) for SH and from 0.31 (Chuannong16) to 0.90 (Wennong5) for RL, respectively (Table 1).

### 2.2. Ranking of Genotypes for Salt Tolerance

Based on the performance of SDW of the examined accessions under salt stress, genotypes with RSDW (salt/control) > 0.7 were identified as salt-tolerant, and those with RSDW < 0.6 were defined as salt-sensitive; those with RSDW of 0.6–0.7 were designated as moderately tolerant (Table S1 and Figure S1). When considering overall measured traits for each genotype, the pooled scores were ranked by the following formula-based integrated relation: integrated score (IS) = absolute values of [(RSDW + RRDW + RSFW + RRFW + RSH + RRL)/6] [27,28]. Based on the IS result, the genotypes with IS > 0.7 were considered salt-tolerant, and those with IS < 0.55 were regarded as salt-sensitive; those with an IS of 0.55–0.7 were defined as moderately tolerant (Table S1). The top four most tolerant genotypes ranked according to RSDW were ranked 3, 2, 1 and 10 according to IS, respectively, and the top four most sensitive genotypes ranked according to RSDW were ranked 334, 329, 326 and 332 according to IS, respectively (Table S1). These results suggest that the two rankings generally agree well with each other, confirming the feasibility of combining the two methods. Finally, a total of 12 wheat genotypes were screened for salt tolerance (genotypes highlighted in yellow in Table S1).

### 2.3. The Confirmatory Experiment

**Figure 1:**
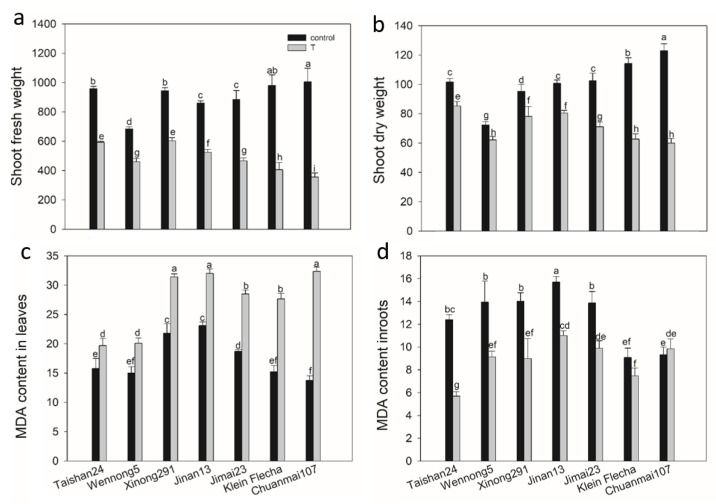
Shoot weight (mg plant^−1^) and malondialdehyde (MDA) content (nmol g^−1^ FW) of seven wheat genotypes under salt stress (T) and control conditions. FW, fresh weight. Data are mean ± STD of eight replicates for shoot fresh weight (**a**) and shoot dry weight (**b**) or three replicates for MDA content in leaves (**c**) and roots (**d**) per genotype and treatment. The different letters mean significant differences among treatments and genotypes according to Duncan’s multiple range, *p* < 0.05.

In the secondary experiment, two sensitive (Klein Flecha and Chuanmai107), one moderately tolerant (Jimai23) and four tolerant (Taishan24, Wennong5, Xinong291 and Jinan13) genotypes were selected based on the screened results to verify the accuracy of the screening. Again, salt stress inhibited plant growth, with reduced SDW and SFW in comparison with those under the control condition (Figure 1a,b). Furthermore, genotypic differences were found in SDW and SFW under salt stress, with the tolerant genotypes having the highest RSDW and RSFW, the sensitive genotypes having lower RSDW and RSFW and the moderately tolerant genotype falling somewhere in between (Figure 1a,b).

**Figure 2:**
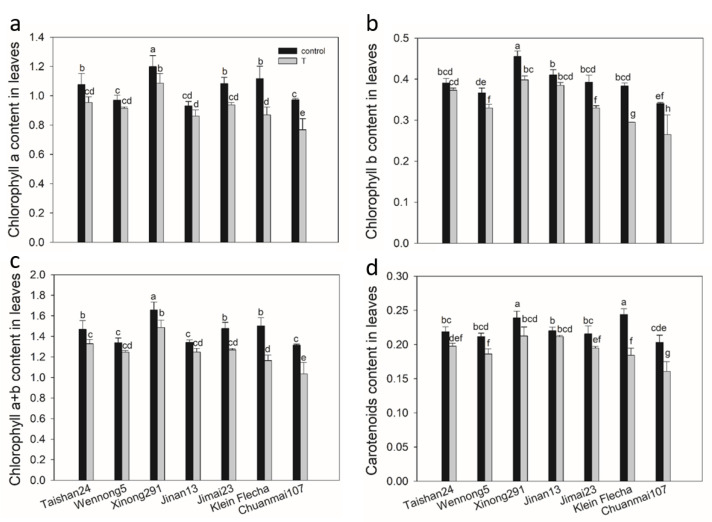
Chlorophyll content (mg g^−1^ FW) of seven wheat genotypes under salt stress (T) and control conditions. (**a**) Chlorophyll a content in leaves under salt stress and control conditions. (**b**) Chlorophyll b content in leaves under salt stress and control conditions. (**c**) Chlorophyll a+b content in leaves under salt stress and control conditions. (**d**) Carotenoids content in leaves under salt stress and control conditions. FW, fresh weight. Data are mean ± STD of three replicates per genotype and treatment. The different letters mean significant difference among treatments and genotypes according to the Duncan’s multiple range, *p* < 0.05.

There was a significant decrease in chlorophyll and carotenoid contents for the salt-sensitive and moderately tolerant accessions, with the reduction reaching more than 20% in the sensitive accessions and 10–16% in the moderately tolerant one under salt stress (Figure 2). For the tolerant accessions subjected to salt stress, no significant change was observed in any of the chlorophylls or carotenoids of Jinan13 or in the chlorophyll a or a+b content of Wennong5, while the other two tolerant accessions displayed a significant but lower decrease in chlorophyll and carotenoid contents compared with salt-sensitive and moderately tolerant accessions (Figure 2). All the tolerant wheat seedlings had less of an effect on chlorophyll and carotenoid contents than the sensitive ones under salt stress, which, in turn, may support their reduced growth inhibition, coinciding with the screening result. Accordingly, Taishan24, Wennong5, Xinong291 and Jinan13 were selected as salt-tolerant accessions, Jimai23 was taken as a moderately tolerant accession, and Klein Flecha and Chuanmai107 were chosen as sensitive accessions for further physiological study.

### 2.4. Accumulation of Osmotic Adjustment Substances

**Figure 3:**
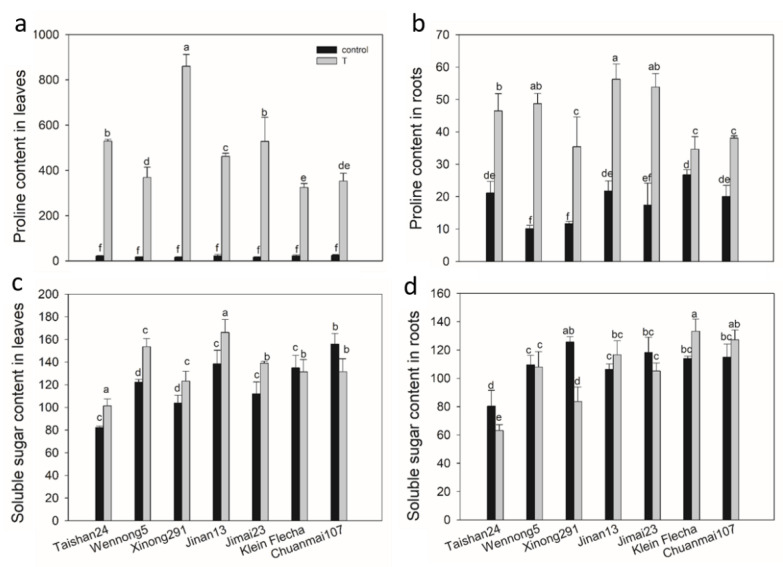
Proline and soluble sugar content (ug g^−1^ FW) of seven wheat genotypes under salt stress (T) and control conditions. (**a**) Proline content in leaves under salt stress and control conditions. (**b**) Proline content in roots under salt stress and control conditions. (**c**) Soluble sugar content in leaves under salt stress and control conditions. (**d**) Soluble sugar content in roots under salt stress and control conditions. FW, fresh weight. Data are mean ± STD of three replicates per genotype and treatment. The different letters mean significant differences among treatments and genotypes according to Duncan’s multiple range, *p* < 0.05.

Salt stress caused a dramatic increase in proline content both in the leaves (324–860 ug g^−1^ FW) and roots (35–56 ug g^−1^ FW) of all seven wheat genotypes compared with the control (16–24 ug g^−1^ FW in leaves and 10–27 ug g^−1^ FW in roots) (Figure 3a,b). Leaf proline content showed greater relative change in comparison with that in roots, with as much as a 13–53-fold increase in leaves and only a 29–380% increase in roots (Figure 3a,b). Moreover, proline in leaves and roots increased to a greater extent in tolerant genotypes than sensitive genotypes under salt stress (Figure 3a,b).

The soluble sugar content exhibited a genotypic and tissue-specific response to salt stress. For leaves, salt stress significantly increased soluble sugar content in the tolerant genotypes, but it reduced or did not change the soluble sugar content in the sensitive genotypes (Figure 3c). In contrast, salt stress significantly decreased or did not change the root soluble sugar content in the tolerant genotypes, but it increased the soluble sugar content in the sensitive genotypes (Figure 3d).

### 2.5. Accumulation of Na and K

Accumulation of Na was found in the shoots of all genotypes under salt stress (Table 2). The highest Na content (69 mg g^−1^ DW) was observed in the sensitive genotype Chuanmai107, while the lowest Na content (25 mg g^−1^ DW) was in the tolerant genotype Taishan24 (Table 2). However, the tolerant genotype Wennong5 also accumulated higher Na content (66 mg g^−1^ DW), which was only significantly lower than that in Chuanmai107 but higher than that in the other genotypes (Table 2). On the other hand, the relative K content was higher in the tolerant genotypes under salt stress, so the tolerant genotypes had lower Na/K ratios than the sensitive genotypes (Table 2).

### 2.6. The Genotypic Difference in MDA Content in Response to Salt Stress

The MDA content exhibited genotypic and tissue-specific differences in response to salt stress among seven accessions (Figure 1c,d). Leaf MDA content showed a dramatic increase under salt stress, increasing by 25, 34, 44, 39, 82, 71 and 135% in Taishan24, Wennong5, Xinong291, Jinan13, Jimai23, Klein Flecha and Chuanmai107, respectively (Figure 1c). By contrast, root MDA content was significantly decreased by 54, 35, 36, 30, 35, and 18% in Taishan24, Wennong5, Xinong291, Jinan13, Jimai23 and Klein Flecha, respectively, while it increased by 6% in Chuanmai107 under salt stress (Figure 1d).

### 2.7. The Genotypic Difference in Antioxidant Enzyme Activity under Salt Stress

**Figure 4:**
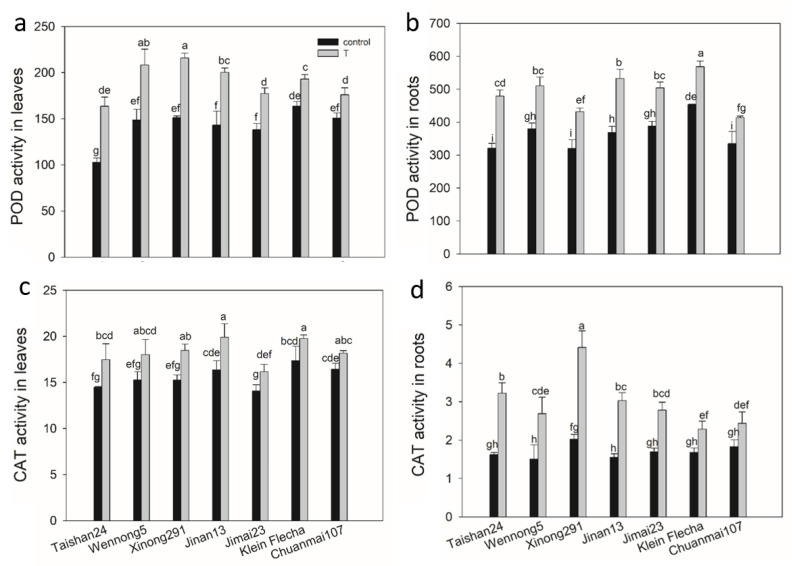
Peroxidase (POD) and catalase (CAT) activity (U g^−1^ FW min^−1^) changes in seven wheat genotypes under salt stress (T) and control conditions. (**a**) POD activity in leaves under salt stress and control conditions. (**b**) POD activity in roots under salt stress and control conditions. (**c**) CAT activity in leaves under salt stress and control conditions. (**d**) CAT activity in roots under salt stress and control conditions. FW, fresh weight. Data are mean ± STD of three replicates per genotype and treatment. The different letters mean significant differences among treatments and genotypes according to Duncan’s multiple range, *p* < 0.05.

**Figure 5:**
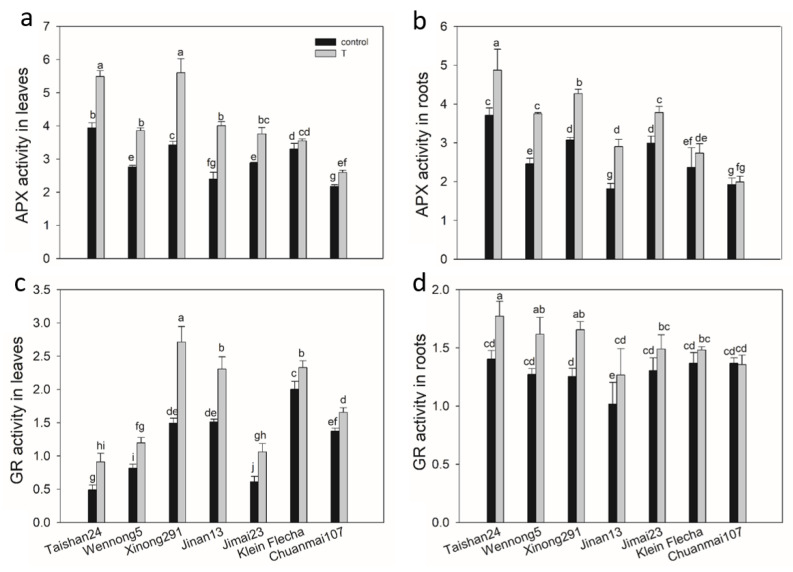
Ascorbate peroxidase (APX) and glutathione reductase (GR) activity (U g^−1^ FW min^−1^) changes in seven wheat genotypes under salt stress (T) and control conditions. (**a**) APX activity in leaves under salt stress and control conditions. (**b**) APX activity in roots under salt stress and control conditions. (**c**) GR activity in leaves under salt stress and control conditions. (**d**) GR activity in roots under salt stress and control conditions. FW, fresh weight. Data are mean ± STD of three replicates per genotype and treatment. The different letters mean significant differences among treatments and genotypes according to Duncan’s multiple range, *p* < 0.05.

The activities of reactive species scavenging enzymes were analyzed for the seven accessions under saline conditions. In generally, the activities of peroxidase (POD), catalase (CAT), ascorbate peroxidase (APX) and glutathione reductase (GR) were significantly increased in all seven accessions, with genotypic and tissue differences under salt stress (Figure 4 and Figure 5). POD activity was higher in roots than in leaves; however, the reverse pattern was observed for CAT activity, with higher CAT activity in leaves than in roots for all genotypes under both treatments (Figure 4). Salt stress increased POD activity by 17–59% in leaves and 24–49% in roots among the seven genotypes, respectively, and the tolerant accessions had greater increases than sensitive accessions (Figure 4). A higher enhancement in CAT activity was also observed in tolerant genotypes than in sensitive genotypes for both leaves and roots under salt stress (Figure 4).

The stimulation of APX activity by salt stress was much higher both in the leaves and roots of tolerant accessions, with the highest increase being 67 and 60% in the leaves and roots of Jinan13, respectively, and the lowest being 7% in the leaves of Klein Flecha and 3% in the roots of Chuanmai107 (Figure 5). A similar pattern was observed for GR activity (Figure 5). Greater relative GR activity in salt stress-tolerant genotypes indicated that the tolerant plants exhibited a more active ascorbate-glutathione cycle than the sensitive genotypes (Figure 5). This cycle has been implicated in the removal of ROS [29].

Comparing the activities of antioxidant enzymes, POD activity was 10-, 110- and 46-fold and 64-, 125- and 55-fold greater in stressed leaves and roots, while it was 9-, 120- and 48-fold greater in leaves and 84-, 111- and 54-fold greater in roots under the control condition, compared with CAT, GR and APX activity, respectively.

## 3. Discussion

### 3.1. Salt Tolerance Estimation for Wheat Genotypes

Plant salt tolerance is growth stage-specific, and salt sensitivity differs at various growth stages [24,30]. In general, cereal plants are the most sensitive to salinity during the vegetative and early reproductive stages [31]. In recent years, the wheat planting area has been increasing in some saline regions of China. It was found that wheat was most sensitive to salt stress at the seedling stage. In other words, this stage is the key stage for selecting salt-tolerant wheat varieties. Here, a set of wheat cultivars, landraces and elite breeding lines from around the world with wide genotypic variation were employed to identify the salt-tolerant and -sensitive accessions at the seedling stage (Table 1).

For crops, more attention should be paid to the grain yield than other traits. In the current study, biomass is the most important parameter related to growth at the seedling stage. Hence, the RSDW of plants was firstly employed to represent salt tolerance [23,32]. However, salt tolerance is a complex biological trait governed by several physiological and genetic factors. To characterize the overall salt tolerance status of wheat genotypes, the IS of the relative change in the measured traits for each genotype was also calculated [27,28]. When the ISs of all examined genotypes were ranked, the tolerant genotypes Taishan24, Wennong5, Xinong291 and Jinan13 had higher values, ranking 3, 2, 1 and 12 among 334 genotypes, respectively (Table S1), suggesting that the combination of the two ranking methods in the current study could be feasible in practice. This screening method takes a combination of one-factor and multifactor analysis, which not only focuses more on the yield but also accounts for the plant growth status.

Chlorophyll content is widely used as an indicator of the abiotic tolerance level, and salinity causes a decrease in chlorophyll content in plants [33,34,35]. The confirmatory experiment showed that the decrease in chlorophylls was lower in all the tolerant accessions in comparison with the sensitive ones (Figure 2). This may indicate that the tolerant accessions suffered less oxidation in chlorophyll and maintained a stable pigment protein complex under salt stress [36], thereby leading to less retarded growth [37]. This is highly consistent with the growth performance under salt stress in the first experiment (Figure 1 and Figure 2), so the second study further confirmed the screening result of the first experiment.

### 3.2. Physiological Analysis among Contrasting Wheat Genotypes

Osmotic stress is the first stress that plants suffer from when exposed to saline soil, and it instantly affects plant growth [38]. Plants can synthesize soluble osmotic adjustment substances to maintain cell turgor and improve osmotic balance at the cellular level to alleviate osmotic stress [10,39,40]. It has been documented that the important osmoprotectants proline and soluble sugars accumulate to high levels in tolerant genotypes under saline conditions [41,42]. In the current study, the tolerant accessions also accumulated much more proline in both leaves and roots and soluble sugars in leaves than that in sensitive plants under salt stress (Figure 3). Greater accumulation of proline and soluble sugars in the tolerant genotypes may help to maintain the osmotic potential and protect plant cells from salt stress. Notably, the root soluble sugar content was up-accumulated in the sensitive genotypes, but it was not changed or down-accumulated in the tolerant genotypes (Figure 3). Soluble sugars can not only stabilize the cell membrane and protoplast [43] but also provide a carbon source and energy for other organic synthesis processes in roots, playing an important role in plant growth [44]. Hence, it may be assumed that the use of soluble sugars in the root of tolerant genotypes was much greater compared to that in the sensitive genotypes under salt stress, contributing to better root growth in the tolerant genotypes.

Salt stress causes ion toxicity in plant cells because of the intracellular ion imbalance caused by a large influx of Na^+^. The tolerant genotypes had the ability to avoid high uptake/accumulation of Na in their shoots, as reported in many studies [22,45]. This easily explains why the highest accumulation of Na occurred in the sensitive genotype Chuanmai107 and the lowest accumulation of Na was in the tolerant genotype Jinan13. However, Na content that accumulated in the tolerant genotype Wennong5 was second only to that in Chuanmai107. Glycophytes cope with salinity stress not only by Na^+^ exclusion from shoots [12,13,14,46] but also by tissue tolerance through the compartmentalization of Na^+^, mainly in vacuoles [13,21,47]. Thus, the different Na contents in the tolerant genotypes may suggest that Na^+^ exclusion and tissue tolerance vary independently. Moreover, different combinations of Na^+^ exclusion and tissue tolerance might contribute to similar levels of salt tolerance [23]. In addition, the higher relative K content in the tolerant genotypes helps to maintain a lower Na/K ratio and ion homeostasis under salt stress, thus conferring salt tolerance [11,14,46,48].

Lipid peroxidation has often been considered an indicator of salt-induced oxidative damage in membranes [17,49]. MDA is a known product of lipid peroxidation, and in the present study, salt-tolerant accessions showed a lower increase in leaf MDA content in comparison with the sensitive accessions under salt stress (Figure 1c). This result indicates that less oxidative damage to cellular membranes was suffered from salt stress in the tolerant accessions: that is to say, tolerant accessions possessed higher cell membrane stability, consistent with previous studies [50,51,52]. The lower relative level of lipid peroxidation in salt-tolerant accessions suggests that better protection from oxidative damage was provided to salt-tolerant accessions. This protection probably results from the more efficient antioxidative system in response to salt stress in salt-tolerant accessions [17,49,53,54]. This is supported by the higher increases in the activities of antioxidative enzymes, including POD, CAT, APX and GR, in the salt-tolerant genotypes.

On the other hand, the MDA content remained unaffected in the roots of Chuanmai107 but decreased dramatically in the roots of other accessions under stress. The reduction in MDA content may be due to increased antioxidative enzyme activities, which reduced ROS levels and membrane damage [49,55,56]. Further studies are necessary for a better and more accurate explanation.

## 4. Materials and Methods

### 4.1. A Preliminary Screening Experiment

The accessions used in the current study consist of 334 diverse wheat genotypes, comprising 274 accessions from China and 60 accessions from 10 other countries, including France (20), Italy (9), Argentina (7), Russia (6), America (5), Japan (4), Romania (6), Australia (1), Hungary (1) and Turkey (1) (Table S1). A hydroponic experiment was carried out in a greenhouse with white fluorescent light (300 μmol m^−2^ s^−1^) at 20 °C under a photoperiod of 16 h light/8 h dark [57,58]. Seeds of all the accessions were germinated in germinating boxes, and then the uniform 7-day-old seedlings were transplanted in 20 L blue containers with 1/2 Hoagland’s solution [59,60] in a completely randomized block design. The container was covered with a polystyrol-plate with 60 evenly spaced holes. The continuously aerated solution was renewed every 5 days [61]. Based on the pre-experiment, two-leaf seedlings (eight for each genotype) were treated with 200 mM NaCl. One week later, SH, RL, SFW, RFW, SDW and RDW were determined with four biological replicates. For each genotype, the average value of two separate plants for each trait was taken as one biological replicate under both salt stress and control conditions.

### 4.2. A Secondary Experiment for Verification

In this experiment, 7 wheat accessions (4 highly tolerant, 1 moderately tolerant and 2 sensitive) were used based on the results of the preliminary experiment. Wheat seedlings were cultivated and treated as described above. Sixteen seedlings were prepared for biomass analysis per genotype and treatment. After 7 days of treatments, seedlings were sampled for the measurement of dry weight, as described above, with eight biological replicates. Chlorophyll and carotenoid contents were determined using fresh samples (200 mg) with three biological replicates according to Lichtenthaler (1987) [62].

### 4.3. Determination of Proline and Soluble Sugar Contents

To analyze the accumulation of osmotic adjustment substances, 200 mg of fresh samples were taken for the determination of proline or soluble sugar content with three replicates in each accession. Free proline content was determined by sulfosalicylic acid assay as described by Bates et al. (1973) [63]. The soluble sugar content was measured with the anthrone-sulfuric acid method according to Giannakoula et al. (2008) [64].

### 4.4. Estimation of Na and K Contents

The dried shoots were finely powdered and sieved (100 mesh) for the determination of Na and K contents in each accession. A 100 mg fine powder was placed in test tubes with 5 mL of a mixture of 60% trichloroacetic acid, nitric acid and sulfuric acid (2:10:1) and then incubated in a 90 °C water bath for 30 min. The supernatant was taken after centrifugation. Na and K contents were estimated using an atomic absorption spectrophotometer (TAS-990).

### 4.5. Measuring of Antioxidant Enzyme Activities and Lipid Peroxidation

For the extraction of antioxidant enzymes, 200 mg of fresh samples were homogenized with 8 mL of extraction buffer (50 mM Na-phosphate buffer, pH 7.8) containing 2% polyvinylpyrrolidone in a chilled mortar on ice. The homogenate was centrifuged at 10,000× *g* for 20 min at 4 °C. The supernatant was used for assaying the activities of enzymes. Three replicates were run.

MDA content was measured according to the method of Kumar and Knowles (1993) [65]. The amount of MDA was expressed as nmol g^−1^ FW. Peroxidase (POD, EC 1.11.1.7) activity was assessed by the method of Hammerschmidt et al. (1982) [66] with modification. One unit of POD activity was defined as the increase in absorbance g^−1^ FW min^−1^ at 470 nm. Catalase (CAT, EC 1.11.1.6) activity was estimated as described by Shi et al. (2007) [67]. One enzyme unit of CAT was indicated by the decline in absorbance g^−1^ FW min^−1^ at 240 nm. Ascorbate peroxidase (APX, EC 1.11.1.11) activity was estimated according to Nakano and Asada (1981) [68]. One unit of enzyme activity was calculated using the decrease in absorbance g^−1^ FW min^−1^ at 290 nm. Glutathione reductase (GR, EC 1.6.4.2) activity was assayed by the method of Wheeler et al. (1990) [69]. One unit of GR was defined as the decrease in absorbance g^−1^ FW min^−1^ at 340 nm.

### 4.6. Statistical Analysis

Significant differences in physiological traits among treatments and genotypes were analyzed using Duncan’s multiple range test (DMRT) in DPS7.05 software, and the differences at *p* < 0.05 and *p* < 0.01 were considered significant and highly significant, respectively. The relative change in each trait was calculated by the value of salt stress/control. The relative changes in SH, RL, SFW, RFW, SDW and RDW were designated as RSH, RRL, RSFW, RRFW, RSDW and RRDW.

### 4.7. Ranking of Genotypes for Salt Tolerance

In the present study, a combination of one-factor (more emphasis on the biomass yield) and multifactor analysis was used to identify salt-tolerant wheat genotypes. For the one-factor analysis, the value of RSDW was used as the index for the ranking of wheat salt tolerance [23,32]. Based on the biomass ranking result, the genotypes with RSDW > 0.7 were considered salt-tolerant, and those with RSDW < 0.6 were defined as salt-sensitive; those with RSDW of 0.6–0.7 were designated as moderately tolerant. For the multifactor analysis, six growth traits (RSDW, RRDW, RSFW, RRFW, RSH, RRL) for each genotype were employed. The pooled scores were ranked, and the formula is as follows: integrated score (IS) = absolute values of [(RSDW + RRDW + RSFW + RRFW + RSH + RRL)/6] [27,28]. Based on the IS result, the genotypes with IS > 0.7 were taken as salt-tolerant, and those with IS < 0.55 were deemed salt-sensitive; those with IS of 0.55–0.7 were defined as moderately tolerant. Only those identified as salt-tolerant genotypes in both ranking methods were considered to be tolerant.

## 5. Conclusions

The wheat genotypes showed considerable variation in measured growth traits under salinity in this study. Thus, these germplasms are suitable for distinguishing salt tolerance among genotypes in response to salt stress. The identified contrasting wheat genotypes showed significantly different physiological responses to salt stress. The tolerant genotypes (Taishan24, Wennong5, Xinong291 and Jinan13) exhibited greater accumulation of osmotic substances, lower shoot Na^+^/K^+^ and higher membrane stability and antioxidative activities than sensitive genotypes (Klein Flecha and Chuanmai107), which might contribute to their better growth under salt stress. The performance of the moderately tolerant genotype (Jimai23) is somewhere in between that of the tolerant and sensitive genotypes. To some extent, it was more similar to the salt-tolerant genotypes in terms of the physiological mechanism. The identified genotypes may be useful for breeding and further genetic studies for salt tolerance.

## Figures and Tables

**Table 1:** Phenotypic variation in salt tolerance-related traits in the 334 wheat accessions.

Trait	Minimum			Maximum		Mean				CV (%)			AmongGenoTypes
	C	T	T/C	C	T	T/C	C	T	T/C	C	T	T/C	
SDW (mg)	83.50	43.00	0.37	213.75	129.75	0.94	145.89	89.09	0.62	16.65	14.38	14.16	**
RDW (mg)	33.00	8.50	0.17	104.50	50.75	1.00	63.71	32.52	0.52	19.75	21.46	21.54	**
SFW (mg)	659.50	231.50	0.22	2069.75	810.00	0.78	1278.17	541.85	0.43	17.66	19.00	17.90	**
RFW (mg)	502.25	131.00	0.14	1759.00	825.25	1.05	1066.10	484.70	0.46	21.41	25.77	26.16	**
SH (cm)	16.50	12.25	0.62	27.40	22.53	1.03	22.02	17.42	0.79	9.75	9.51	7.54	**
RL (cm)	23.58	7.73	0.31	48.73	29.58	0.90	34.71	21.22	0.62	12.33	13.96	13.48	**

Note: C, control; T, salt stress; T/C, ratio of each trait value between salt stress and control; SDW, shoot dry weight; RDW, root dry weight; SFW, shoot fresh weight; RFW, root fresh weigh; SH, shoot height; RL, root length; **, highly significant differences among genotypes according to Duncan’s multiple range, *p* < 0.01, n = 4.

**Table 2:** Phenotypic differences in K and Na content (mg g^−1^ DW) in seven wheat accessions.

Genotype	K Content			Na Content	Na/K
	C	T	T/C	T	T
Taishan24	69.97h	68.46i	0.98	24.67f	0.36
Wennong5	71.36g	85.70a	1.20	66.11b	0.77
Xinong291	71.53g	74.98de	1.05	37.79d	0.50
Jinan13	74.58ef	73.35f	0.98	32.50e	0.44
Jimai23	76.03cd	78.84b	1.04	38.06d	0.48
Klein Flecha	69.65hi	62.15j	0.89	52.50c	0.84
Chuanmai107	76.43c	59.43k	0.78	68.88a	1.16

Note: C, control; T, salt stress; T/C, ratio of each trait value between salt stress and control. DW, dry weight. For each trait, different lowercase letters indicate significant differences among the treatments and genotypes according to Duncan’s multiple range, *p* < 0.05, n = 3.

## Data Availability

The available data are contained within the article.

## Supplementary Materials

Supplementary materials can be found at https://www.mdpi.com/2223-7747/10/2/268/s1. Table S1: The 334 wheat accessions used in the screening study for salt tolerance, their origins and growth performance under salt stress. Figure S1: The distribution of relative shoot dry weight (a) and integrated score (b) of 334 wheat genotypes in the preliminary selection experiment.

## Abbreviations

APX: ascorbate peroxidase; C: control; CAT: catalase; GR: glutathione reductase; IS: integrated score; MDA: malondialdehyde; POD: peroxidase; RDW: root dry weight; RFW: root fresh weight; RL: root length; RRDW: relative change of root dry weight; RRFW: relative change of root fresh weight; RRL: relative change of root length; RSDW: relative change of shoot dry weight; RSFW: relative change of shoot fresh weight; RSH: relative change of shoot height; SDW: shoot dry weight; SFW: shoot fresh weight; SH: shoot height; T: salt stress; ROS: reactive oxygen species.
