# Supplementary material for: Identification and Characterization of Wheat Germplasm for Salt Tolerance
Source: Plants (Basel). 2021 Jan 30;10(2):268. doi: 10.3390/plants10020268 (PMC7911706; doi:10.3390/plants10020268)
Supplement: Supplementary file 1 [file plants-10-00268-s001.zip › plants-1091908-supplementary/supplementary files/Figure S1.docx]

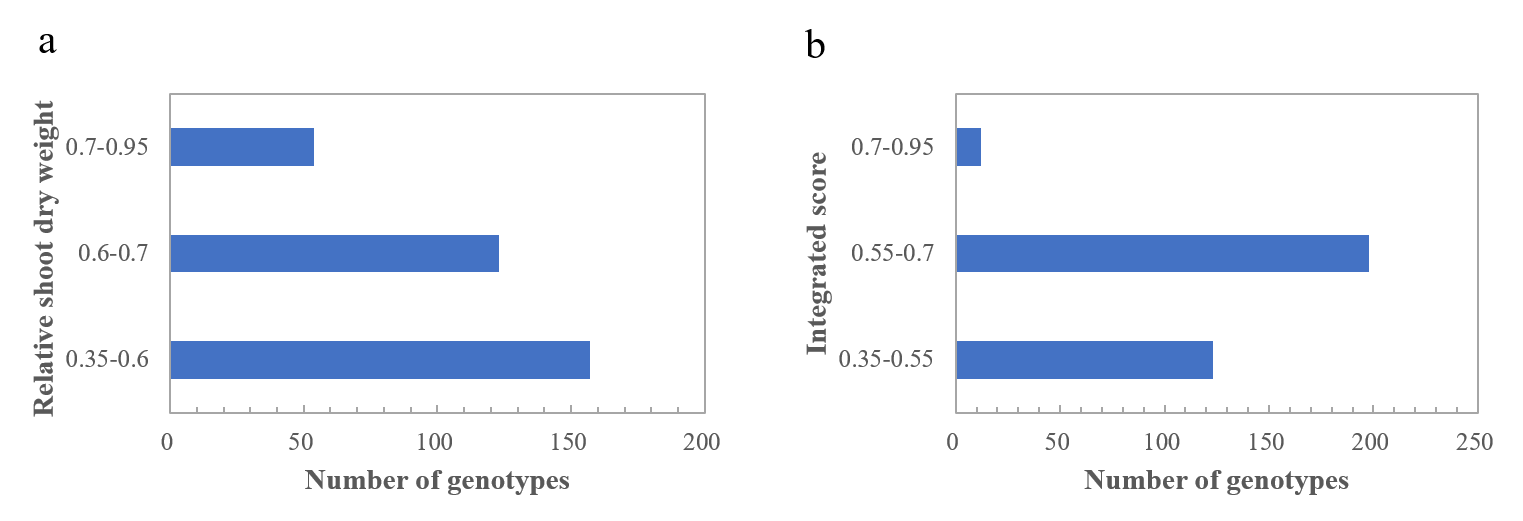


Figure S1 The distribution of relative shoot dry weight (a) and integrated score (b) of 334 wheat genotypes in the preliminary selection experiment.
